# Combining grain yield, protein content and protein quality by multi-trait genomic selection in bread wheat

**DOI:** 10.1007/s00122-019-03386-1

**Published:** 2019-07-01

**Authors:** Sebastian Michel, Franziska Löschenberger, Christian Ametz, Bernadette Pachler, Ellen Sparry, Hermann Bürstmayr

**Affiliations:** 1 Department of Agrobiotechnology, IFA-Tulln, University of Natural Resources and Life Sciences Vienna, Konrad-Lorenz-Str. 20, 3430 Tulln, Austria; 2 Saatzucht Donau GesmbH & CoKG, Saatzuchtstrasse 11, 2301 Probstdorf, Austria; 3 C&M Seeds, 6180 5th Line, Palmerston, ON N0G 2P0 Canada

## Abstract

**Key message:**

Simultaneous genomic selection for grain yield, protein content and dough rheological traits enables the development of resource-use efficient varieties that combine superior yield potential with comparably high end-use quality.

**Abstract:**

Selecting simultaneously for grain yield and baking quality is a major challenge in wheat breeding, and several concepts like grain protein deviations have been developed for shifting the undesirable negative correlation between both traits. The protein quality is, however, not considered in these concepts, although it is an important aspect and might facilitate the selection of genotypes that use available resources more efficiently with respect to the quantity and quality of the final end products. A population of 480 lines from an applied wheat breeding programme that was phenotyped for grain yield, protein content, protein yield and dough rheological traits was thus used to assess the potential of using integrated genomic selection indices to ease selection decisions with regard to the plethora of quality traits. Additionally, the feasibility of achieving a simultaneous genetic improvement in grain yield, protein content and protein quality was investigated to develop more resource-use efficient varieties. Dough rheological traits related to either gluten strength or viscosity were combined in two separate indices, both of which showed a substantially smaller negative trade-off with grain yield than the protein content. Genomic selection indices based on regression deviations for the two latter traits were subsequently extended by the gluten strength or viscosity indices. They revealed a large merit for identifying resource-use efficient genotypes that combine both superior yield potential with comparably high end-use quality. Hence, genomic selection opens up the opportunity for multi-trait selection in early generations, which will most likely increase the efficiency when developing new and improved varieties.

**Electronic supplementary material:**

The online version of this article (10.1007/s00122-019-03386-1) contains supplementary material, which is available to authorized users.

## Introduction

The genetic improvement in grain yield is a major breeding goal in bread wheat, whose achievement is, though, oftentimes complicated by large genotype-by-environment interaction and a complex quantitative inheritance governed by many small-to-medium effect quantitative trait loci (QTL) (Hoffstetter et al. 2016b; Schulthess et al. 2017). Its low heritability and complex inheritance render grain yield, thus an interesting target trait for the application of genomic selection (Crossa et al. 2017). The prediction of genotype performance in yet untested years is, however, still challenging, even with a genomic breeding approach (Hoffstetter et al. 2016a; Jarquin et al. 2017; Huang et al. 2018; Juliana 2018) and its merit furthermore dependent on the respective breeding programme (Rife et al. 2018). Nevertheless, the application of genomic selection has been shown to lead to a similar or higher genetic gain in comparison with early-generation phenotypic selection in a conventional breeding scheme (He et al. 2016; Michel et al. 2017; Belamkar et al. 2018) and has been implemented into many national and international breeding programmes (Lado et al. 2016; Cericola et al. 2017; Fiedler et al. 2017; Guzmán et al. 2017).

The maintenance of or improvement in end-use quality is on the other hand mostly a secondary objective in many bread wheat breeding programmes, and an indirect selection for baking quality traits is often conducted by using the protein content as a major selection criterion in early generations. Aside from the protein content, end-use quality in wheat is determined by the protein quality that can among others be assessed with dough rheological tests (Branlard et al. 1992; Anderssen et al. 2004; Schiedt et al. 2013). The numerous dough rheological traits are generally less influenced by genotype-by-environment interactions in comparison with the protein content (Williams et al. 2008; Hernández-Espinosa et al. 2018) but involve on the other hand labour-intensive, time-consuming and costly quality analysis. Hence, an early-generation selection for these baking quality-related traits can either be conducted indirectly by using small-scale tests (Knott et al. 2009; Guzmán et al. 2016; Malegori et al. 2017) or by employing genomic selection directly targeting the underlying genetic architecture. The prediction accuracy of a genomic selection approach can be further refined by upweighting known major QTL or the inclusion of prior information of correlated traits like the protein content or sedimentation value into prediction models (Hayes et al. 2017; Lado et al. 2018; Michel et al. 2018). A plethora of information with regard to baking quality-related traits is thus made available when implementing genomic selection in early generations. Although this might generally be considered as a beneficial feature, it also gives rise to the challenging task of identifying the lines with the desired combination of these dough rheological traits. Additionally, breeders might aim to develop high-quality genotypes with acceptable yield potential or develop high-yielding varieties while maintaining quality characteristics, both of which are complicated by the frequently observed negative correlation between grain yield and the major quality criterion protein content (Simmonds 1995).

Notwithstanding, selection for both breeding goals can be conducted either by employing grain protein or yield deviations (Rapp et al. 2018; Thorwarth et al. 2018; Michel et al. 2019; Thorwarth et al. 2019) with the former being closely related to yield-adjusted breeding values for protein content (Hänsel 2001; Arief et al. 2010). Both concepts can furthermore be seen as an application of restriction indices holding either the protein content or grain yield stable and improving the other trait. A simultaneous selection for both traits can also be based on protein yield that is, though, stronger correlated with grain yield than protein content (McNeal 1982; Simmonds 1995). Alternatively, the mentioned concepts can be combined by high yield and protein indices which aim to achieve a high protein yield either via an elevated grain yield or protein content (Michel et al. 2019). The derived selection indices are moreover associated with nitrogen-use-efficiency-related traits like post-anthesis nitrogen uptake and remobilization (Monaghan et al. 2001; Bogard et al. 2010) and have shown some potential to mitigate the protein content/grain yield trade-off by compensating for the dilution of the protein content when selecting for higher yield potential (Michel et al. 2019). Nevertheless, the protein quality is not considered in these indices yet, although it is an important aspect when breeding for both grain yield and baking quality as seen by some wheat hybrids that possess a lower protein content, though, a higher yield potential and similar sedimentation value as line varieties (Thorwarth et al. 2018). Genotypes that combine superior grain yield with comparably high-quality characteristics putatively utilize available resources more efficiently with respect to the quantity and quality of the final end product like baked breads. Hence, extending the existing concepts for a simultaneous selection of grain yield and protein content by the inclusion of protein quality seems necessary to facilitate the development of varieties with a more effective combination of yield potential and end-use quality. The usage of selection indices has a large potential to ease selection decisions in this endeavour with regard to the plethora of involved traits (Smith 1936; Hazel and Lush 1942), while genomic selection indices (Togashi et al. 2011; Ceron-Rojas et al. 2015) have in this case the particular advantage that they can already be employed in early generations before high-quality phenotypic data are available. The aims of this study were thus (i) to assess the potential of using integrated selection indices to ease selection decisions with regard to protein quality traits and (ii) investigate the feasibility to achieve a simultaneous genetic improvement in grain yield and baking quality-related traits in order to develop more resource-use efficient varieties.

## Materials and methods

### Plant material and phenotypic data

This study focused on the analysis of a diverse population of 480 F_4:6_ generation and double haploid winter wheat breeding lines (*Triticum aestivum* L.) from an applied breeding programme that were developed from 394 families and tested in multi-environment trials under Central and Eastern European conditions from 2009 to 2016. The phenotypic data comprised information about their grain yield (dt ha^−1^), protein content (%), protein yield (dt ha^−1^) and multiple dough rheological traits dough rheological parameters from the Extensograph and Farinograph. The data were obtained from multiple partially connected trial series with a total of 156 trials and more than 2000 lines (Table S1). A set of 480 lines was chosen due to the availability of completely orthogonal phenotypic records for the numerous traits of interest, where grain yield, protein content and protein yield were tested in several series of 136, 86 and 66 preselected trials with sufficient data quality, respectively (see next section). The trials were on average connected by 57, 90 and 54 checks as well as F_4:6_ and F_7_ breeding lines within years, but the connection across years was mainly established by around 48, 41 and 46 selected F_4:6_ lines that were retested in the F_7_ the subsequent year. Each line was on average replicated seven times for grain yield and five times for protein content and protein yield.

The dough rheological traits were on the other hand available from 29 preselected trials, laid out as completely randomized trial designs that were on average connected by 43 lines within years and 13 lines across all years. Each line was on average replicated three times for the assessment of dough rheology which for most lines corresponded to testing in three different trials, while within trials only 1–3 checks were generally tested in replication. These traits were regarded as measures for differentiating lines for their protein quality in this study. Their assessment is generally costly, labour-intensive as well as time-consuming and involved as a first-step milling the collected grain samples with a Quadrumat Junior milling system according to method AACC26-50 of the American Association of Cereal Chemists (AACC, 2000). Dough mixing properties were subsequently determined by a Farinograph (Brabender GmbH and Co KG) equipped with a 300-g mixing bowl, in which the water uptake of each flour sample was estimated in a pretest on a 100-g subsample until it reached an optimal dough consistency of 500 farinogram units (FU) according to the standard procedure AACCI 54e21 (AACC, 2000). The main test with intensive mixing included the measurement of the dough development time as the time frame in minutes from the first water uptake until the dough began to soften as well as the dough stability that was quantified as the time in minutes between the first intersection and leaving of the 500 FU reference line by the kneading curve. Additionally, dough softening was evaluated as the difference in observed dough consistency and the 500 FU reference line after 12 min of kneading, i.e. lower values are more favourable; however, for convenience, for example, to achieve a positive correlation with the other dough mixing parameters the phenotypic records for dough softening were inverted by multiplying them with -1 for all subsequent analysis. Viscoelastic properties of the flour samples were thereafter determined by the Extensograph (Brabender GmbH and Co KG) according to AACCI 54-10.01 (AACC, 2000). Hence, the dough rheological profile of each line was completed by embedding the extensibility (mm), resistance to extension at 50 mm in Extensogram units (EU) and the area under the Extensograph curve, i.e. the dough energy (cm^2^), after a 135-minute resting time.

### Statistical analysis of phenotypic data

Phenotypic data from all original 156 multi-environment trials were firstly analysed with various models correcting for spatial trends. Briefly, a baseline model without spatial correction was compared with all 15 possible combinations of random row and/or column effects with/without modelling a variance–covariance structure between the plots either in row, in column or in both directions by an autoregressive spatial model (AR1) (Burgueño et al. 2000) The model with the best fit was subsequently chosen by Akaike’s information criterion (AIC). Best linear unbiased estimates (BLUE) were derived with this model, and the heritability was estimated with $h^{2} = {{\sigma_{G}^{2} } \mathord{\left/ {\vphantom {{\sigma_{G}^{2} } {\left( {\sigma_{G}^{2} + \frac{1}{2}{\text{MVD}}} \right)}}} \right. \kern-0pt} {\left( {\sigma_{G}^{2} + \frac{1}{2}{\text{MVD}}} \right)}}$, where $\sigma_{G}^{2}$ designates the genetic variance and ${\text{MVD}}$ the mean variance of a difference in the BLUEs (Piepho and Möhring 2007). Multi-environment trials with a heritability larger than 0.3 were forwarded to an across-trial analysis for grain yield, protein content and protein yield, while for the rheological traits all trials with a heritability smaller than 0.1 instead of 0.3 were excluded from further analysis. This liberal threshold was chosen due to the costly, labour-intensive as well as time-consuming process for assessing these traits. Additionally, when estimating the heritability for dough rheological traits within trials, the error variance was estimated based on the replicated checks making the strong assumption that the other lines have the same error variance. These estimates were thus regarded as a coarse indication about the data quality of the individual trials, while the across-trial analysis was seen as being the relevant measure of data quality for the dough rheological data. Furthermore, in some trials none of the lines were tested in replicate for dough rheology; thus, the data could not be assessed for its quality in these cases but were still integrated into the analysis due to the mentioned circumstances. All traits were subsequently analysed with a linear mixed model of the form:

1 $$y_{ij} = \mu + g_{i} + t_{j} + r_{ij}$$

where $y_{ij}$ are the BLUEs for the respective trait from the first stage, $\mu$ is the grand mean and $g_{i}$ is the effect of the *i*th line. The effect of the *j*th trial $t_{j}$ was fixed, while the effect $r_{ij}$ that incorporated both the trial-by-line interaction variance and the residual effect was assumed random and followed a normal distribution with ${\mathbf{r}} \sim N\left( {0, {\mathbf{I}}\sigma_{r}^{2} } \right)$. The heritability of the across-trial analysis was again computed by $h^{2} = {{\sigma_{G}^{2} } \mathord{\left/ {\vphantom {{\sigma_{G}^{2} } {\left( {\sigma_{G}^{2} + \frac{1}{2}{\text{MVD}}} \right)}}} \right. \kern-0pt} {\left( {\sigma_{G}^{2} + \frac{1}{2}{\text{MVD}}} \right)}}$. Additionally, a genomic heritability was estimated for the entire set of 480 lines that was used for genomic prediction in this study with details being reported in the following sections. All phenotypic analyses were conducted using the statistical package ASReml for the R programming environment (R Development Core Team 2018).

### Genotypic data and population structure

Leaf samples were collected from a minimum of ten plants from each F_4:5_ or doubled haploid line and used for DNA extraction with the protocol given by Saghai-Maroof et al. (1984). The DArT genotyping-by-sequencing (GBS) approach (Diversity Arrays Technology Pty Ltd) was used for genotyping all 480 lines, and quality control was applied by filtering with regard to a call rate lower than 90% and removing markers with a minor allele frequency smaller than 0.05 as well as more than 10% of missing data. A subset of 457 lines was furthermore screened for their allelic state at the high molecular weight glutenin subunit loci *Glu*-*A1*, *Glu*-*B1* and *Glu*-*D1* by sodium dodecyl sulphate–polyacrylamide gel electrophoresis (SDS–PAGE). The allele calls from this analysis were recoded into an − 1, 0, + 1 format to facilitate their integration into the genomic relationship matrix for genomic prediction. The allele less favourable 0 for the *Glu*-*A1* was coded as − 1, while the favourable alleles 1 and 2* were both coded as + 1 due to their similar effect (Payne et al. 1987). The allele 6 + 8 of the *Glu*-*B1* locus was coded as -1 and the alleles 7 + 8 as well as 7 + 9 were coded as +1 again due to their similar effect. The alleles 5 + 10 and 2 + 12 of the *Glu*-*D1* locus were finally coded as − 1 and + 1, respectively, while heterozygous marker alleles were generally coded as 0. The missForest algorithm (Stekhoven and Bühlmann 2012) was subsequently used for a chromosome-wise imputation of missing data points for the remaining 7.3 K SNP markers. The average modified Rogers’ distance in the population was calculated as $D_{\text{MR}} = 0.31$. The population structure with the corresponding membership of each line to its subpopulation as well as their allelic state at the *Glu*-*1* loci was finally investigated by principal component analysis (Suppl. Fig. S1; Suppl. Fig. S2).

### Single-trait genomic prediction models

The merit of genomic prediction for grain yield, protein content and protein quality as well as possible negative trade-offs was investigated in a resampling scheme, where 300 lines were 100 times randomly sampled in training population and 100 lines in validation population. The kinship between lines was for this purpose estimated by the genomic relationship matrix, which was computed according to the method described by Endelman and Jannink (2012) with the *M* = 7.3 K SNP markers:

2 $${\mathbf{K}} = {\mathbf{WW}}^{\text{T}} /2\sum {\left( {p_{k} - 1} \right)p_{k} }$$

where ${\mathbf{W}}$ is a centred NxM marker matrix of the N lines with $W_{ik} = Z_{ik} + 1 - 2p_{k}$ with $p_{k}$ being the allele frequency at the kth locus and $Z_{ik}$ the marker allele of the *i*th line at the kth locus. Genomic estimated breeding values (GEBV) were afterwards derived by genomic best linear unbiased prediction models (GBLUP) including the obtained genomic relationship matrix:

3 $${\mathbf{y}} = 1_{\varvec{N}} \mu + {\mathbf{Z}}_{{\mathbf{G}}} {\mathbf{u}}_{{\mathbf{G}}} + {\mathbf{r}}$$

where ${\mathbf{y}}$ is an Nx1 vector of BLUEs obtained in the phenotypic analysis, ${\mathbf{Z}}_{{\mathbf{G}}}$ a random-effect design matrix for additive genetic effects and ${\mathbf{u}}_{{\mathbf{G}}}$ is an Nx1 vector of additive effects with ${\mathbf{u}}_{{\mathbf{g}}} \sim N\left( {0, {\mathbf{K}}\sigma_{{u_{G} }}^{2} } \right)$. The residual effect ${\mathbf{r}}$ followed a normal distribution ${\mathbf{r}} \sim N\left( {0, {\mathbf{I}}\sigma_{r}^{2} } \right)$, and $\mu$ designates the intercept with $1_{N}$ being a Nx1 vector where all elements equal 1. The potential of exploiting prior information of known major QTL for baking quality-related traits was furthermore investigated by extending the previous model to a weighted genomic best linear unbiased prediction (WBLUP) that included the SDS–PAGE markers for the *Glu*-*1* loci as separate effects (Bernardo 2014; Zhao et al. 2014; Arruda et al. 2016; Spindel et al. 2016; Michel et al. 2018a):

4 $${\mathbf{y}} = 1_{\varvec{N}} \mu + {\mathbf{Z}}_{{\mathbf{G}}} {\mathbf{u}}_{{\mathbf{G}}} + {\mathbf{M}}_{{{\mathbf{A}}1}} \beta_{{{\text{A}}1}} + {\mathbf{M}}_{{{\mathbf{B}}1}} \beta_{{{\text{B}}1}} + {\mathbf{M}}_{{{\mathbf{D}}1}} \beta_{{{\text{D}}1}} + {\mathbf{r}}$$

where ${\mathbf{y}}$ is an Nx1 vector of BLUEs obtained in the phenotypic analysis and ${\mathbf{u}}_{{\mathbf{G}}}$ is an Nx1 vector of additive effects as beforehand. The marker effects $\beta_{{{\text{A}}1}}$, $\beta_{{{\text{B}}1}}$ and $\beta_{{{\text{D}}1}}$ for the *Glu*-*A1*, *Glu*-*B1* and *Glu*-*D1* locus were modelled either as fixed or as random, with the latter aiming to achieve a differential marker effect shrinkage in concordance with the explained variance of each marker for the according dough rheological trait. The appropriate coding for the marker alleles at the corresponding *Glu*-*1* loci was for this purpose modelled by the three Nx1 vectors ${\mathbf{M}}_{{{\mathbf{A}}1}}$, ${\mathbf{M}}_{{{\mathbf{B}}1}}$ and ${\mathbf{M}}_{{{\mathbf{D}}1}}$. Hence, in the case of the GBLUP model the genomic breeding value of each line was estimated by the formula:

5 $${\text{GEB}}\;V_{i} = \mu + g_{i}$$

which was extended in the WBLUP model to:

6 $${\text{GEB}}\;V_{i} = \mu + g_{i} + \sum\limits_{j = 1}^{n} {m_{ij} \beta_{j} }$$

with $g_{i}$ being the random genetic effect of the *i*th line, $\mu$ the grand mean, $\beta_{j}$ the estimated effect of the *j*th *Glu*-*1* loci marker and $m_{ij}$ the marker allele of the *i*th line at the *j*th *Glu*-*1* loci marker. The merit of genomic prediction was additionally compared with the possibility of a marker-assisted prediction using merely the *Glu*-*1* loci either as fixed or as random predictors with fitting the additive genetic effect ${\mathbf{u}}_{{\mathbf{G}}}$ in model (4). All models for genomic prediction were fitted with the mixed model package *sommer* (Covarrubias-Pazaran 2016) for R (R Development Core Team 2018).

### Genomic selection indices for grain yield, protein content and protein quality

**Fig. 1:**
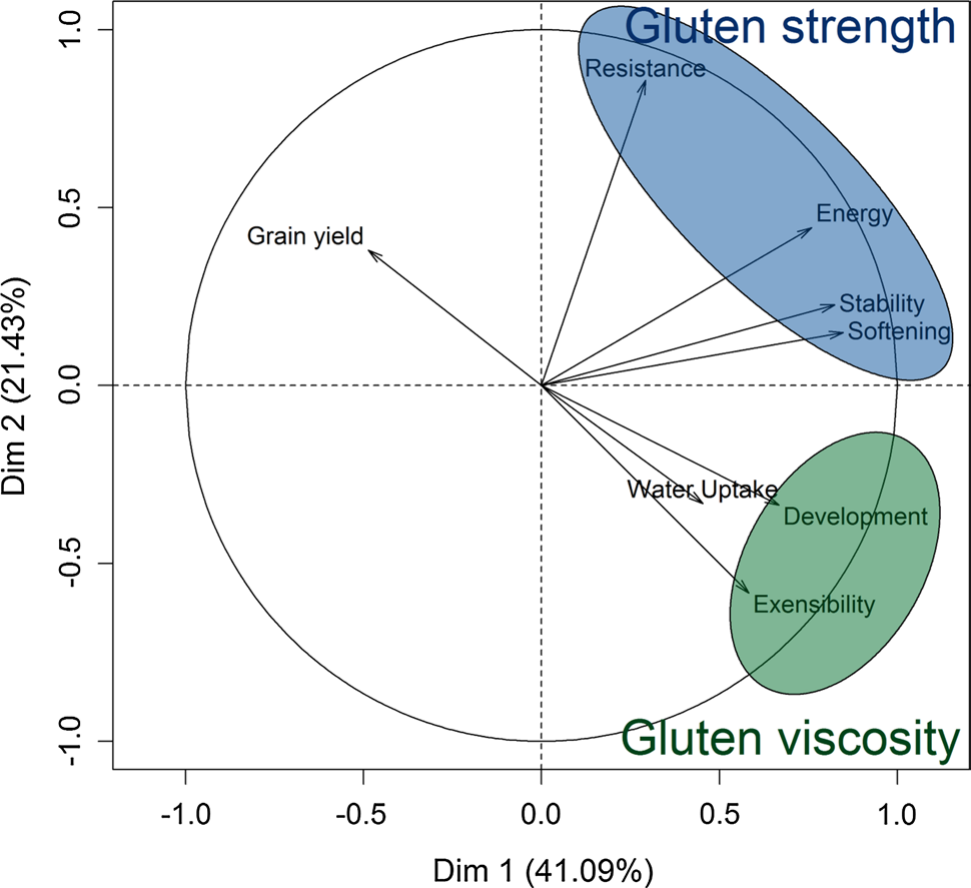
Relationship between grain yield and dough rheological traits within the set of 480 lines, highlighting the designated gluten strength and gluten viscosity groups, where the latter contained only the dough development and extensibility

The possibility to summarize the plethora of dough rheological traits into integrated quality indices to ease selection decisions was subsequently investigated by using the same resampling scheme with 100 replicates described in the previous section. Based on the loadings of a principal component analysis with the 480 lines, two major groups of correlated traits could be identified in accordance with previous studies (Maphosa et al. 2015; Marti et al. 2015; Huen et al. 2018; Lado et al. 2018) (Fig. 1), and they were designated as the gluten viscosity and gluten strength groups in this study. Although a strict differentiation of the trait variation causing gluten strength and viscosity is not feasible using dough rheological parameters, especially in the presence of interactions (Weipert 2006), two separate indices that aimed to largely target these two aspects of protein quality were derived from this grouping:

7 $${\text{Index}}_{{{\text{STRH}}_{i} }} = {\text{RES}}_{i} {\text{b}}_{\text{RES}} + {\text{ENG}}_{i} {\text{b}}_{\text{ENG}} + {\text{STAB}}_{i} {\text{b}}_{\text{STAB}} + {\text{SOFT}}_{i} {\text{b}}_{\text{SOFT}}$$

where the gluten strength index (${\text{Index}}_{{{\text{STRH}}_{i} }}$) for the *i*th line was calculated by combining genomic breeding values obtained from single-trait genomic predictions using the corresponding index weights for the extensogram resistance to extension $b_{\text{RES}}$ and dough energy $b_{\text{ENG}}$ as well as for the dough stability $b_{\text{STAB}}$ and softening $b_{\text{SOFT}}$ from the farinogram. These index weights were obtained from a genomic selection index of the form:

8 $${\mathbf{b}} = {\mathbf{G}}^{ - 1} {\mathbf{a}}$$

with ${\mathbf{b}}$ being a vector of index weights, ${\mathbf{a}}$ the vector of desired gains and ${\mathbf{G}}^{ - 1}$ the inverse of the genomic variance–covariance matrix based on centred and standardized genomic estimated breeding values from single-trait predictions of the respective traits:

9 $$\left( {\begin{array}{*{20}l} {\sigma_{\text{RES}}^{2} } \hfill & \sigma \hfill & \sigma \hfill & \sigma \hfill \\ \sigma \hfill & {\sigma_{\text{ENG}}^{2} } \hfill & \sigma \hfill & \sigma \hfill \\ \sigma \hfill & \sigma \hfill & {\sigma_{\text{STAB}}^{2} } \hfill & \sigma \hfill \\ \sigma \hfill & \sigma \hfill & \sigma \hfill & {\sigma_{\text{SOFT}}^{2} } \hfill \\ \end{array} } \right)$$

with the variances of the genomic estimated breeding values for the dough rheological trait on diagonal and covariance between traits on the off-diagonal. The vector of desired gains was set to ${\mathbf{a}} = \left( {a_{\text{RES}} = 1,\;a_{\text{ENG}} = 1,\;a_{\text{STAB}} = 1,\;a_{\text{SOFT}} = 1} \right)^{\text{T}}$, which corresponded to an equal weighting of the four dough rheological traits. The gluten viscosity index was analogously defined by:

10 $${\text{Index}}_{{{\text{VISC}}_{i} }} = {\text{EXT}}_{i} b_{\text{EXT}} + {\text{DEV}}_{i} b_{\text{DEV}}$$

and comprised the combination of the individual genomic breeding values by index weights for extensibility $b_{\text{EXT}}$ and dough development time $b_{\text{DEV}}$ derived from (8) with the genomic variance–covariance matrix

11 $$\left( {\begin{array}{*{20}l} {\sigma_{\text{EXT}}^{2} } \hfill & \sigma \hfill \\ \sigma \hfill & {\sigma_{\text{DEV}}^{2} } \hfill \\ \end{array} } \right)$$

with the vector of desired gains ${\mathbf{a}} = \left( {a_{\text{EXT}} = 1,\;a_{\text{DEV}} = 1} \right)^{\text{T}}$. The water uptake showed generally a lower phenotypic correlation with the other dough rheological traits and was regarded as standing alone (Suppl. Fig. S3).

The main interest of this study was nevertheless to investigate the feasibility to achieve a simultaneous genetic improvement in grain yield, protein content and protein quality by genomic prediction. Genomic index selection for this purpose was firstly based on regression residuals from a regression of protein content on grain yield that are commonly known as grain protein deviations (Monaghan et al. 2001; Bogard et al. 2010; Rapp et al. 2018; Thorwarth et al. 2018, 2019). This protein residual method has been shown to be equivalent to a restriction index selection holding grain yield stable while increasing the protein content (Michel et al. 2019) and was extended in this study to:

12 $${\text{Index}}_{{{\text{GPD}}_{i} }} = {\text{PC}}_{i} b_{\text{pc}} + {\text{GY}}_{i} b_{\text{gy}} + {\text{Index}}_{{{\text{STRH}}_{i} }} b_{\text{STRH}} + {\text{Index}}_{{{\text{VISC}}_{i} }} b_{\text{VISC}}$$

which contained aside from the genomic breeding values based on single-trait predictions for protein content ${\text{PC}}_{i}$ and grain yield ${\text{GY}}_{i}$ and also the gluten viscosity ${\text{Index}}_{{{\text{VISC}}_{i} }}$ and strength index values ${\text{Index}}_{{{\text{STRH}}_{i} }}$ of the *i*th line. All index weights were again obtained from formula (8) with appropriate modifications of the genomic variance–covariance matrix ${\mathbf{G}}$ after centring and standardizing the genomic estimated breeding and index values of all involved traits, while the desired gains were set to ${\mathbf{a}}_{{{\mathbf{GPD}}}} = \left( {a_{\text{pc}} = 1,\;a_{\text{gy}} = 0,\;a_{\text{STRH}} = \lambda ,\;a_{\text{VISC}} = \gamma } \right)^{\text{T}}$ with $\gamma$ and $\lambda$ varying in the interval $\left[ {0,1} \right]$. The analogous extension of grain yield deviation that aim to hold the protein content stable while increasing grain yield (Rapp et al. 2018; Michel et al. 2019) gave rise to the mirror-inverted picture by altering the vector of desired gains to ${\mathbf{a}}_{{{\mathbf{GYD}}}} = \left( {a_{\text{pc}} = 0,\;a_{\text{gy}} = 1,\;a_{\text{STRH}} = \lambda ,\;a_{\text{VISC}} = \gamma } \right)^{\text{T}}$ in order to derive an index of the form:

13 $${\text{Index}}_{{{\text{GYD}}_{i} }} = {\text{GY}}_{i} b_{\text{gy}} + {\text{PC}}_{i} b_{\text{pc}} + {\text{Index}}_{{{\text{STRH}}_{i} }} b_{\text{STRH}} + {\text{Index}}_{{{\text{VISC}}_{i} }} b_{\text{VISC}}$$

Additionally, a selection for increased protein yield was conducted in the form of a high yield index using the deviations from regressing protein yield on protein content, which aimed at the identification of lines that show a superior protein yield due to a high grain yield potential (Michel et al. 2019):

14 $${\text{Index}}_{{{\text{HY}}_{i} }} = {\text{PY}}_{i} b_{\text{py}} + {\text{PC}}_{i} b_{\text{pc}} + {\text{Index}}_{{{\text{STRH}}_{i} }} b_{\text{STRH}} + {\text{Index}}_{{{\text{VISC}}_{i} }} b_{\text{VISC}}$$

with ${\text{PY}}_{\text{i}}$ being the genomic breeding value for the protein yield of the *i*th line and $b_{\text{py}}$ the corresponding index weight. The complementary high protein index of the *i*th line was calculated by:

15 $${\text{Index}}_{{{\text{HP}}_{i} }} = {\text{PY}}_{i} b_{\text{py}} + {\text{GY}}_{i} b_{\text{gy}} + {\text{Index}}_{{{\text{STRH}}_{i} }} b_{\text{STRH}} + {\text{Index}}_{{{\text{VISC}}_{i} }} b_{\text{VISC}}$$

and was intended to detect outstanding lines that possess an elevated protein yield due to their performance in protein content. Economic weights for the high yield and protein indices were given by ${\mathbf{a}}_{{{\mathbf{HY}}}} = \left( {a_{\text{py}} = 1,\;a_{\text{pc}} = 0,\;a_{\text{STRH}} = \lambda ,\;a_{\text{VISC}} = \gamma } \right)^{\text{T}}$ and ${\mathbf{a}}_{{{\mathbf{HP}}}} = \left( {a_{\text{py}} = 1,\;a_{\text{gy}} = 0,\;a_{\text{STRH}} = \lambda ,\;a_{\text{VISC}} = \gamma } \right)^{\text{T}}$ with $\gamma$ and $\lambda$ varying again in the interval $\left[ {0,1} \right]$.

### Prediction accuracy and response to selection

The prediction ability was assessed by correlating the genomic breeding and index values with observed values for grain yield, protein content, protein yield and all dough rheological parameters. The prediction accuracy was subsequently calculated as the prediction ability divided by the square root of the heritability. For this purpose, a genomic heritability was estimated for each trait within each of the 100 times randomly resampled validation population by:

16 $$h_{\text{GEN}}^{2} = {{\left( {\sigma_{P}^{2} - \sigma_{e}^{2} } \right)} \mathord{\left/ {\vphantom {{\left( {\sigma_{P}^{2} - \sigma_{e}^{2} } \right)} {\sigma_{p}^{2} }}} \right. \kern-0pt} {\sigma_{p}^{2} }}$$

where $\sigma_{P}^{2}$ is the phenotypic variance of investigated trait and $\sigma_{e}^{2}$ the error variance obtained from a GBLUP model that only contained phenotypic data from the validation population. It should be mentioned that Eq. (16) was also used to compute a genomic heritability to measure the quality of the phenotypic data for the entire set of 480 lines used in this study (Table S2). Aside from assessing the prediction accuracy, it was of further interest to investigate how these estimates would translate into a response to selection. The 10% best performing lines among the 100 selection candidates of each resampling step were therefore selected according to the above-described genomic selection indices, the phenotypic values within the validation population were centred and standardized, and a predicted response measured in standard deviations was computed by:

17 $$\widehat{R}_{{{\text{Sel}}_{i} }} = \mu_{i} + h_{{{\text{GEN}}_{i} }}^{2} \left( {\mu_{{{\text{Sel}}_{i} }} - \mu_{i} } \right)$$

where $\mu_{i}$ is the average trait performance of an entire validation population, $\mu_{{{\text{Sel}}_{i} }}$ is the average trait performance of the selected lines and $h_{{{\text{GEN}}_{i} }}^{2}$ is the genomic heritability of the *i*th trait. The predicted response of each selection method by index combination was finally recorded for all traits that were investigated in this study.

## Results

### Prediction accuracy for single-trait genomic breeding values and quality indices

The prediction accuracy for the dough rheological traits was high and varied between *r* = 0.646 for resistance to extension up to *r* = 0.728 for water uptake in the basic GBLUP model for genomic prediction (Suppl. Table S2). The average prediction accuracy for genomic prediction (*r* = 0.710) was moreover substantially higher than a marker-assisted prediction using only the three *Glu*-*1* loci (*r* = 0.449). However, some gain in average prediction accuracy was realized by integrating the major *Glu*-*1* loci markers as separate fixed (*r* = 0.725) or random (*r* = 0.727) effects into the genomic prediction models. The advantage of such WBLUP models with random *Glu*-*1* marker effects was especially pronounced for the dough stability (+ 2.3%), dough energy (+ 4.8%) and the resistance to extension (+ 9.4%) that are well known to be strongly influenced by the *Glu*-*1* loci (Oury et al. 2010; Michel et al. 2018). The other major agronomic traits were as expected hardly influenced by upweighting the *Glu*-*1* marker effects and a generally high prediction accuracy could be achieved with a basic GBLUP model for grain yield (*r* = 0.751) and the protein content (*r* = 0.769), while it was lower for the protein yield (*r* = 0.671).

**Fig. 2:**
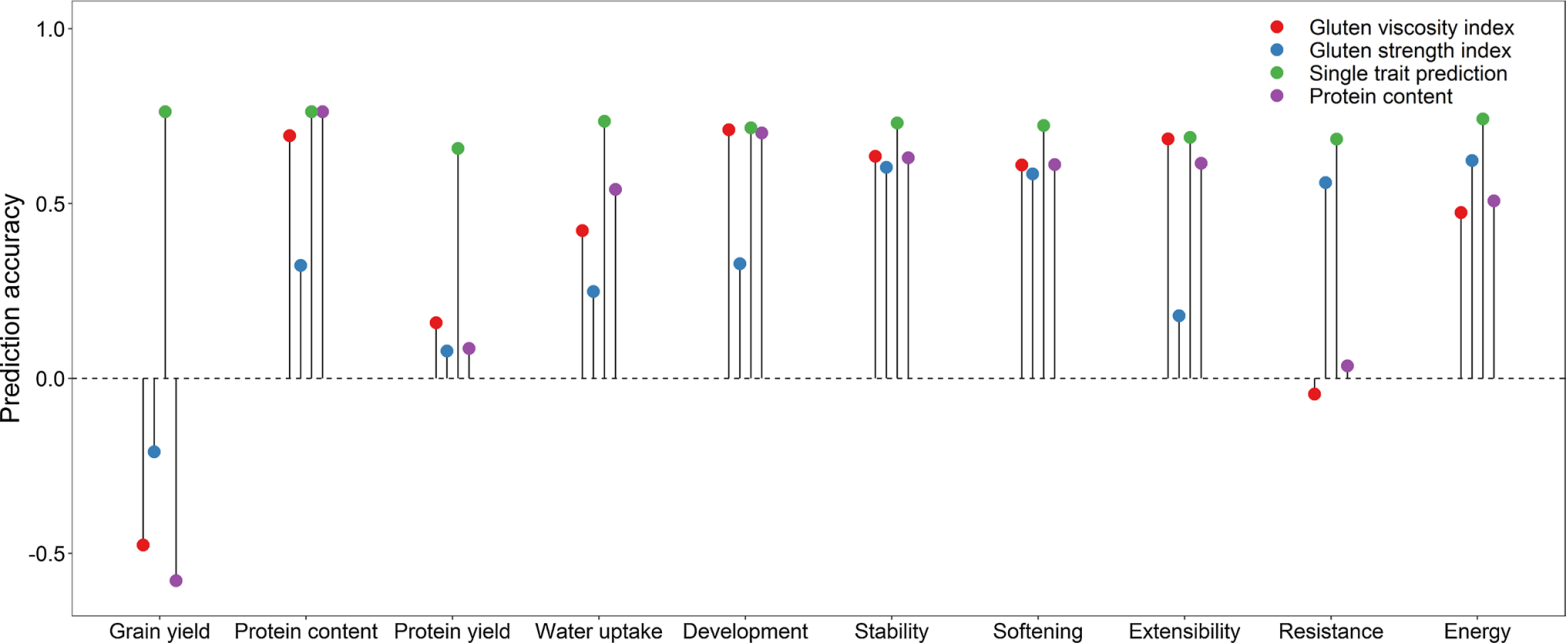
Prediction accuracy for grain yield, protein content and protein yield as well as the dough rheological traits when using genomic estimated breeding values of the traits per se, protein content or the gluten strength and viscosity index for prediction

Aiming to ease selection decisions with the plethora of dough rheological traits related to protein quality, the potential of integrating them into several protein quality indices was subsequently investigated by introducing a grouping according to their phenotypic correlation (Fig. 1; Suppl. Fig. S3). The prediction accuracy of the gluten viscosity index amounted to *r* = 0.685 for the extensibility and *r* = 0.710 for dough development, while traits like the resistance to extension in the gluten strength group were on average, as expected, much less accurate predicted when employing this index (*r* = 0.418) (Fig. 2). The mirror-inverted picture could consequently be observed when utilizing the gluten strength index for genomic prediction. It should, though, be noted that the prediction accuracy of single-trait predictions per se instead of genomic index combinations was always higher for the dough rheological traits. The same observation was made for the selection of the best 10% of lines, where the response to index selection for the dough rheological traits was, though, always higher than using the protein content as an indirect predictor trait (Suppl. Fig. S4).

It was evident that the trade-off between grain yield and the gluten strength index that encompassed major rheological traits like dough energy and stability was much lower in terms of prediction accuracy (*r* = − 0.210) than the grain yield/protein content trade-off (*r* = − 0.578). A similar observation was made for the gluten viscosity index albeit its trade-off with grain yield was larger (*r* = − 0.478) in comparison with the gluten strength index, suggesting that an simultaneous improvement in grain yield and baking quality might be more readily achieved by targeting dough rheology, i.e. protein quality instead of the protein content.

### Simultaneous genomic selection for grain yield, protein content and protein quality

**Fig. 3:**
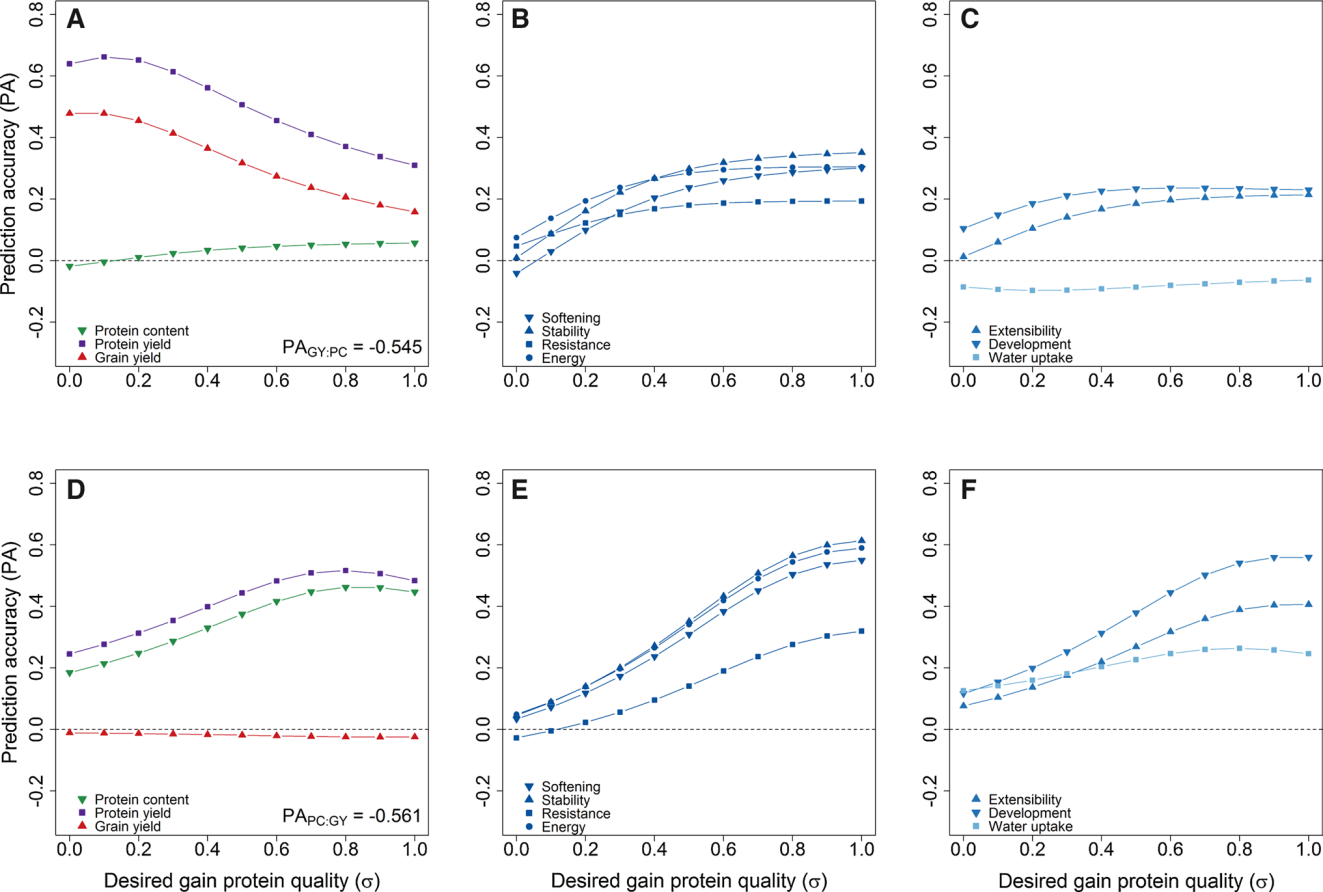
Prediction accuracy for major agronomic traits, gluten strength- and gluten viscosity-related dough rheological traits as well as the water uptake with varying desired gains for the protein quality in the high yield (**a**–**c**) and high protein indices (**d**–**f**)

The merit of genomic selection for developing high-yielding varieties, while maintaining quality characteristics, was subsequently tested by augmenting the previously described high yield index (Michel et al. 2019) with the gluten strength and viscosity indices. The high yield index generally aimed to increase the protein yield while holding the protein content stable, which led a positive prediction accuracy for both grain and protein yield irrespective of the desired gain for the protein quality, i.e. gluten strength and viscosity (Fig. 3a). Aiming for larger gains in protein quality generally reduced, though, the prediction accuracy for grain yield from *r* = 0.478 to *r* = 0.158, i.e. when keeping protein quality stable or giving equal weight on both grain yield and protein quality. The prediction accuracy for dough rheological traits increased analogously, albeit only marginally with desired gains larger than σ = 0.5 (Fig. 3b, c). An optimum prediction accuracy for the protein yield was achieved (*r* = 0.662) with ${\mathbf{a}}_{{{\mathbf{HY}}}} = \left( {a_{\text{py}} = 1,\;a_{\text{pc}} = 0,\;a_{\text{STRH}} = 0.1,\;a_{\text{VISC}} = 0.1} \right)^{\text{T}}$, at which the prediction accuracy for grain yield (*r* = 0.478) and protein content (*r* = − 0.004) indicated that the actual goal of the high yield index of holding the protein content stable could be fulfilled. Noticeably, the prediction accuracy of all rheological traits expect water uptake was also positive at this optimum point, underpinning the previous statement about the simultaneous improvement in grain yield and baking quality via targeting the protein quality. Genomic selection of high-quality genotypes with acceptable yield potential seemed likewise feasible, where the optimal prediction accuracy for protein yield was achieved with ${\mathbf{a}}_{{{\mathbf{HP}}}} = \left( {a_{\text{py}} = 1,\;a_{\text{gy}} = 0,\;a_{\text{STRH}} = 0.8,\;a_{\text{VISC}} = 0.8} \right)^{\text{T}}$ (Fig. 3d). The prediction accuracy for the protein yield, protein content and all dough rheological traits was accordingly positive (*r* = 0.389–0.565) (Fig. 3e, f), whereas a small negative prediction accuracy was found for grain yield (*r* = − 0.024) that still represented a strong adjustment by the high protein index given the large negative trade-off between protein content and grain yield.

**Fig. 4:**
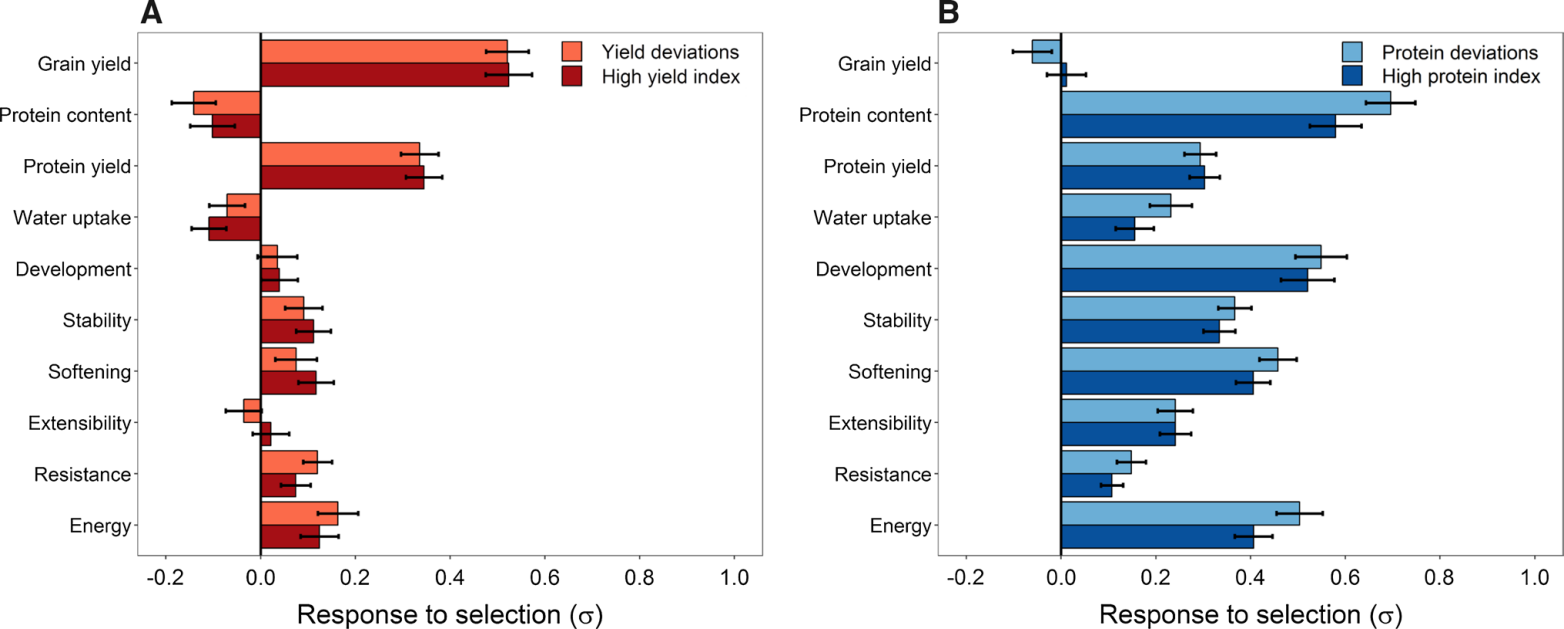
Response to selection of the 10% best performing lines with the high yield and grain yield deviation indices (**a**) as well as the high protein and grain protein deviation indices (**b**). The desired gains that maximized the prediction accuracy and thus putatively also the response to selection for protein yield were chosen for the gluten strength- and gluten viscosity-related dough rheological traits separately for each of the indices

Similar patterns were observed for genomic selection indices based on yield and grain protein deviations (Suppl. Fig. S5); their optimal prediction accuracy for protein yield was, though, slightly lower in comparison with the high yield and protein indices. This issue was also reflected by the higher response to selection for protein yield by the latter two indices (Fig. 4), which generally performed better for compensating the grain yield/protein content trade-off. A direct selection on grain yield resulted in a large positive response for this trait in terms of standard deviations (Δ_GY_ = 0.75) but at the same in a substantial diminishment of the protein content (Δ_PC_ = − 0.55) and dough rheological quality (Δ_RHEO_ = − 0.32). Selection of the 10% best performing lines with the high yield index and previously determined optimal weights mitigated the negative response both for the protein content (Δ_PC_ = − 0.10) and dough rheological traits (Δ_RHEO_ = 0.05), while a positive response to selection could be achieved for all latter traits expect the water uptake. It went moreover along with a much larger response for protein yield (Δ_PY_ = 0.34) than a direct selection on grain yield alone (Δ_PY_ = 0.16). Analogously, a direct selection on protein content (Δ_PC_ = 1.12) clearly reduced grain yield (Δ_GY_ = − 0.54), which was accompanied by a severely lower response to protein yield (Δ_PY_ = 0.05) in comparison with a genomic selection with the high protein index (Δ_PY_ = 0.30) that also facilitated a substantial selection response for all dough rheological traits (Δ_RHEO_ = 0.31).

## Discussion

This study concentrated on genomic breeding methods for conducting a simultaneous selection for grain yield, protein content and dough rheological traits related to baking quality in an applied winter wheat breeding programme. Integrated genomic selection indices were for this purpose compared among each other to develop varieties that combine superior yield potential with comparably high end-use quality and ease selection decisions in general.

### Protein quantity and quality

The protein content is currently a major payment criterion for wheat farmers and millers and is commonly used as quality benchmark for precursors of pasta and bakery products like flour or semolina (Laidig et al. 2018; Rapp et al. 2018). The protein composition is apart from the protein content of pivotal importance, as it significantly determines the properties of bakery product like frozen dough, vital gluten or the cooking quality of pasta (Marti et al. 2013; Frauenlob et al. 2017; Ortolan and Steel 2017). Hence, both the protein content and composition with respect to the gliadin and glutenin protein fractions are of high relevance when developing new cultivars as seen by a larger amount of explained variance of the loaf volume in baking tests when combining the protein content with the sedimentation value in a common prediction model (Laidig et al. 2018). It has been accordingly suggested to rethink the usage of the protein content as the sole quality measure in wheat, especially as numerous varieties with comparably low protein content can produce good bread volume and are accordingly severely underrated when priced merely by their protein content (Gabriel et al. 2017; Zörb et al. 2018).

Various tests are available for assessing traits related to protein quality in wheat, among which the assessment of viscoelastic dough properties by the Farinograph, Extensograph or Alveograph has gained some popularity and is routinely conducted in mills, in bakeries and by breeders. Such dough rheological tests are of special interest to the latter for conducting a pre-selection for quality characteristics, as baking tests are most times too costly for a large number of breeding lines. Nevertheless, dough rheological tests even in small-scale formats can often themselves be time-consuming and labour-intensive, and the widely used unreplicated testing makes a reliable selection difficult in early generations due to genotype-by-environment interactions (Mkhabela et al. 2018; Sapirstein et al. 2018). Implementing genomic selection with a large number of genome-wide distributed markers is thus an attractive alternative for practical breeding programmes (Battenfield et al. 2016; Fiedler et al. 2017; Hayes et al. 2017; Kristensen et al. 2018; Michel et al. 2018). The effect of major QTL associated with gliadins and glutenin and their corresponding genetic and biochemical markers is, though, largely underestimated in the commonly used GBLUP models for genomic selection of quality traits, where among others the *Glu*-*1* loci are of specifically high importance for bread making (Payne 1987). Including the associated markers as separate effects into genomic prediction models resulted consequently in some increase in prediction accuracy in the study at hand. Nonetheless, it has also to be considered that lines fixed for many seemingly favourably *Glu*-*1* alleles can possess unfavourable baking quality characteristics such as too strong and stiff dough characteristics causing a low suitability for bread baking (Ito et al. 2011, 2015). However, modelling major QTL as separate effects in genomic prediction models rapidly increases the frequency of the putatively favourable alleles within a few cycles of genomic selection (Bernardo 2014) warranting a careful handling of the corresponding genomic breeding values for dough rheological traits in practical applications.

Selection decisions that are supported by genomic predictions should thus be appropriately adapted to the respective market demands in the target region of a plant breeding programme. Finding lines with desired trait combinations that satisfy these specific market demands is nevertheless inherently difficult. Selection indices can in this respect be valuable tools to ease selection decisions and achieve a simultaneous response to selection for multiple traits given among others the plethora of dough rheological traits related to protein quality. Despite the complex relationships that exist between these traits, groups of more closely correlated dough rheological traits could be identified. This suggests that traits within these groups are targeting diverse aspects of protein quality that can be partly attributed to different protein fractions. The resistance of extension is, for example, rather associated with glutenin and gluten strength, whereas the extensibility has a closer connection with the gliadin protein fraction and gluten viscosity. The prediction accuracy of the gluten strength and viscosity indices varied accordingly dependant on the predicted dough rheological trait. The resistance to extension could thus not be predicted by the gluten viscosity index, whereas the dough stability that was assigned to the gluten strength group could also be predicted by the gluten viscosity index. A clear-cut differentiation into protein fractions is consequently not feasible by dough rheological traits as several interactions between the glutenin and gliadin protein fractions influence traits like the dough stability or development, which are among others dependant on the glutenin/gliadin ratio (Weipert 2006). Notwithstanding, the particular worth of the suggested indices in comparison with traditional single-point measures like the sedimentation value is given by considering aside from the actual index values also the multiple single-trait predictions. This possibility might give a markedly higher confidence to the undertaken selection decisions since both the benefits and the deficits of a selection candidate can be investigated across an array of diverse traits. Lastly, index weights that aim at a general improvement in dough rheological traits were computed for simplicity in this study, although for some quality traits intermediate values might be more desirable to provide a suitable dough quality (Lado et al. 2017). Hence, the usage of further more sophisticated indices that allow a selection for intermediate trait values should be envisaged (Itoh and Yamada 1988), which could subsequently be combined with major agronomic traits like protein content and grain yield.

### Multi-trait genomic selection for grain yield and baking quality

Grain yield is a primary breeding goal in cereals, and a large genetic improvement in yield potential has been achieved in bread wheat during the last decades (Graybosch and Peterson 2010; Cormier et al. 2013; Sanchez-Garcia et al. 2013; Laidig et al. 2017), oftentimes by breeding for a higher number of grains per unit area for which enhancing spikelet fertility, i.e. number of grains per spikelet, has most likely played a major role (Würschum et al. 2018). The improvement in quality is on the other hand mostly a secondary breeding goal and the protein content as one major baking quality-related trait suffered on average a reduction by continuous wheat breeding (Sanchez-Garcia et al. 2015; Laidig et al. 2017) or was at least kept stable in the last 50 years (Morgounov et al. 2013; Guzmán et al. 2017). The strong negative correlation between grain yield and protein content represents one possible reason for this trend, which led, though, in combination with the mentioned progress in grain yield mostly to an increased protein yield, i.e. total seed nitrogen yield in modern wheat varieties (Cormier et al. 2013). Despite this seeming stagnation in quality breeding, a large genetic progress for baking quality has been made over the years by increasing loaf volume, attaining more favourable rheological characteristics and balances between rheological parameters (Morgounov et al. 2013; Sanchez-Garcia et al. 2015; Guzmán et al. 2017).

The observed improvement in baking quality can thus mostly be attributed to protein quality that additionally showed a much lower trade-off with grain yield than the protein content in this study, suggesting that a simultaneous improvement in baking quality and grain yield is quite feasible. Similar observations have also been made for other crop products like tofu where selection of soybean lines with acceptable tofu quality and superior seed yield seems feasible, despite a negative association of the latter trait with the protein content (Kurasch et al. 2018). The here presented selection strategy that aimed to increase grain yield while maintaining quality characteristics reflected accordingly the overall long-term trend of phenotypic selection in bread wheat breeding. It has furthermore been shown by classical studies that the usage of selection indices is more effective than either independent culling or tandem selection (Hazel and Lush 1942; Pesek and Baker 1969) for such a multi-trait selection. Nevertheless, the necessary information for index selection is often not available or incomplete in early generations of variety development in conventional breeding programmes, which will most likely lead to a higher efficiency

Hence, the implementation of genomic selection into a breeding programme opens up the opportunity for index, i.e. multi-trait selection in early generations. Given that objective index weights for this purpose are difficult to derive, desired gain and especially restriction indices were investigated for supporting breeders in a combined genomic selection for grain yield, protein content and protein quality. The suggested high yield index was able to achieve the goal of increasing the grain yield while holding the protein stable, whereas the simultaneous improvement in dough rheological traits associated with gluten viscosity fraction like extensibility and dough development turned out to be still challenging. These difficulties might be caused by the close relationship between the total protein content, gluten viscosity and gliadin which can alter the glutenin/gliadin ratio in an unfavourable way with regard to dough rheology (Wieser and Kieffer 2001). An example in this study is given by the dough extensibility that showed the strongest negative correlation with grain yield among all dough rheological traits, which caused a slight negative response to selection in genomic index selection with yield deviations. Obtaining a simultaneous gain for grain yield and dough rheological traits in the gluten strength group was on the other hand readily feasible. Notwithstanding, as mentioned above, traits within the designated groups such as dough stability and softening are also strongly influenced by interactions between the protein fractions that are conferred by disulphide bounds, hydrogen bounds and non-covalent interactions between gliadins and glutenins (Dobraszczyk 2004; Wieser 2007). On the other hand, there is also a market demand for high-quality varieties that have an acceptable yield potential. This breeding goal can be addressed during selection by employing either grain protein deviations (Rapp et al. 2018; Thorwarth et al. 2018, 2019) or protein yield (McNeal 1982) as selection criteria. Alternatively, a combination of the mentioned concepts in an index aiming to achieve a high protein yield either via an elevated protein content can be beneficial (Michel et al. 2019). This concept was extended by the here suggested gluten strength and viscosity indices, and the corresponding high protein index was able to mitigate the grain yield/protein content trade-off, while it facilitated at the same time a substantial response to selection for protein quality and protein yield.

The here presented methods for genomic selection could thus enable an earlier and more efficient shifting of the undesired correlation between grain yield and baking quality in bread wheat. They could furthermore be applied in both variety development and population improvement in general, for example, when using a two-part strategy with a rapid recurrent genomic selection and separate product development cycle (Gaynor et al. 2017; Gorjanc et al. 2018). The index weights for protein quality need, however, to be adapted to the respective goals of a wheat breeding programme as, for example, aiming to increase both grain yield and protein quality could be desirable, but the presented results suggested that holding the latter stable might also be a convenient option. Finally, it should also be mentioned that replacing the two rheological indices for gluten strength and viscosity by genomic breeding values for baking volume in the combined indices with grain yield, protein content and protein yield can also be an interesting alternative given that a large enough training population was phenotyped for this trait. Such a strategy might be highly desirable, as the selection of genotypes that possess a higher baking quality relative to their grain yield has recently been proposed as a complementing option when breeding for nitrogen use efficiency (Hawkesford 2014; Cormier et al. 2016) aside from traits like post-anthesis nitrogen uptake and remobilization (Monaghan et al. 2001; Bogard et al. 2010; Lammerts van Bueren and Struik 2017).

## Conclusions

This study investigated the potential and limits of genomic selection indices to facilitate a simultaneous selection for grain yield and baking quality-related traits for breeding putatively more resource-use efficient varieties. The suggested genomic selection indices revealed a large merit for identifying genotypes that combine both superior yield potential with comparably high end-use quality and the development of varieties with high baking quality while preserving a sufficient amount of grain yield. Genomic selection indices could thus be regarded as valuable decision-making tools that should, however, be combined with breeders’ observations, experience and knowledge about germplasm within and beyond a breeding programme. The development of resource-use efficient varieties by breeders can furthermore be seen as one important component in finding sustainable solutions for the challenges that modern agricultural systems are currently facing; to fully harness their potential in a wheat-to-bread supply chain including breeders, extension services, farmers, millers and food processing must, though, be involved (Goucher et al. 2017).

### Author contribution statement

SM wrote the manuscript and analysed the data. CA supported in the statistical analysis. FL, BP and ES designed the field trials and collected the phenotypic data in the field. FL and HB initiated and guided through the study. All authors read and approved the final manuscript.

## Electronic supplementary material

Below is the link to the electronic supplementary material.

Supplementary material 1 (PDF 1195 kb)
