# Supplementary material for: Combining grain yield, protein content and protein quality by multi-trait genomic selection in bread wheat
Source: Theor Appl Genet. 2019 Jul 1;132(10):2767–80. doi: 10.1007/s00122-019-03386-1 (PMC6763414; doi:10.1007/s00122-019-03386-1)
Supplement: Supplementary file 1 — Supplementary material 1 (PDF 1195 kb) [file 122_2019_3386_MOESM1_ESM.pdf]

Supplementary material

Article Title: Combining grain yield, protein content and protein quality by multi-trait genomic selection in bread wheat

Journal: Theoretical and Applied Genetics

Authors: Sebastian Michel, Franziska Löschenberger, Christian Ametz, Bernadette Pachler, Ellen Sparry, Hermann Bürstmayr

Name, affiliation, and email of corresponding author:

Sebastian Michel
Department for Agrobiotechnology (IFA-Tulln)
Institute for Biotechnology in Plant Production
University of Natural Resources and Life Sciences, Vienna (BOKU)
Konrad-Lorenz-Str. 20, 3430 Tulln, Austria
e-mail: sebastian.michel@boku.ac.at

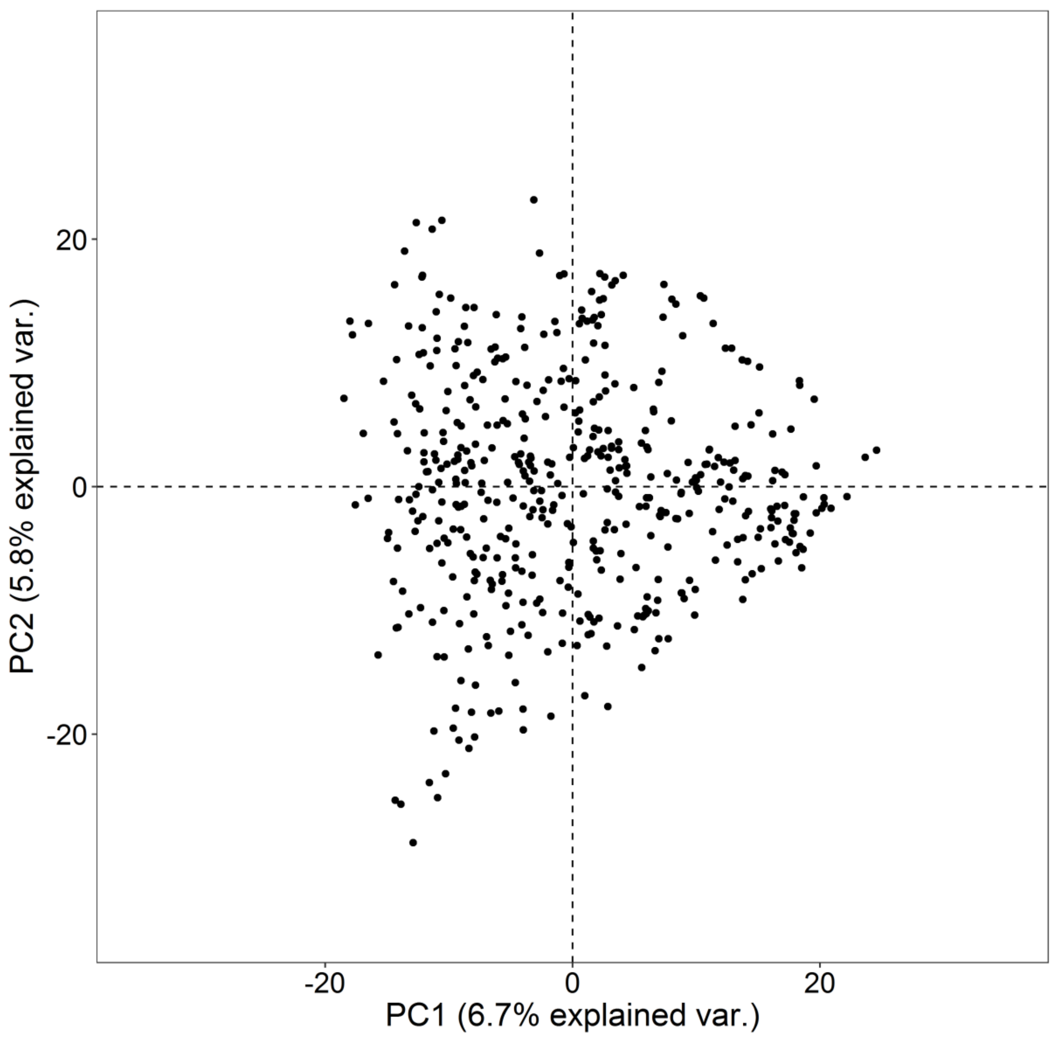

Fig. S1 Population structure of the 480 lines involved in the study.

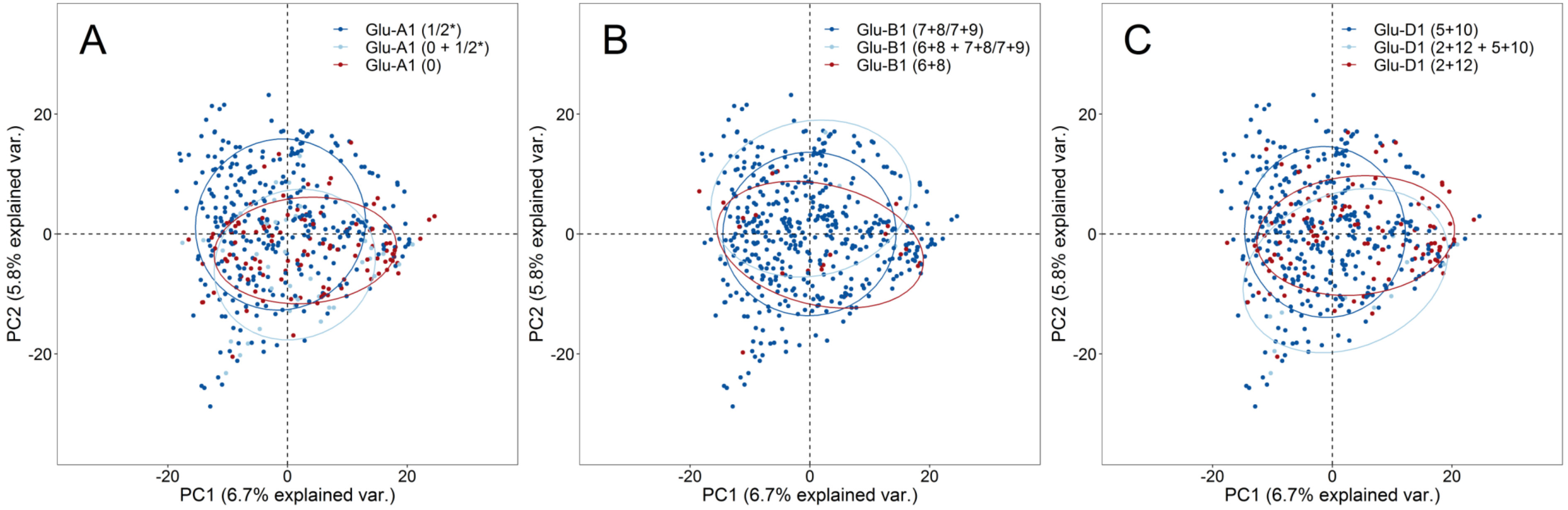

Fig. S2 Population structure of the 480 studied lines with respect to their alleles at the Glu-A1 (A), Glu-B1 (B), and Glu-D1 (C) marker locus.

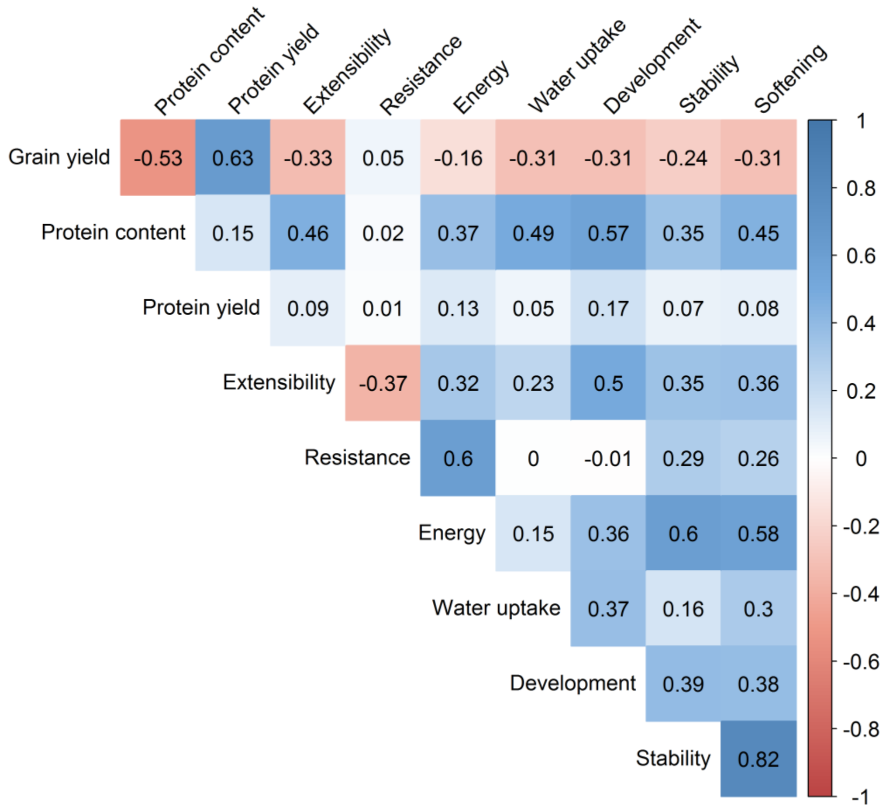

Fig. S3 Phenotypic correlations between grain yield, protein content, protein yield and the dough rheological traits for the 480 studied lines. The phenotypic values for dough softening were inverted to achieve a positive correlation with the other dough mixing parameters.

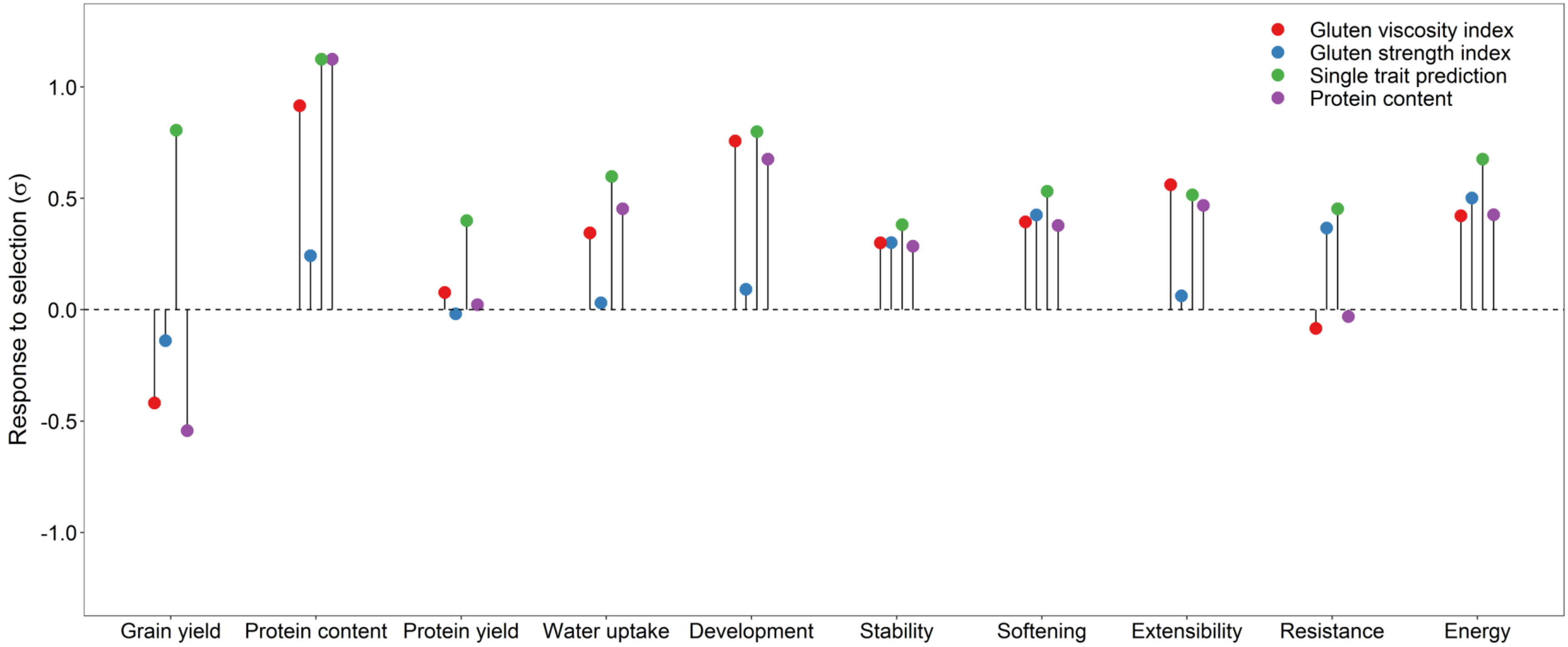

Fig. S4 Response to selection of the 10% best performing lines for grain yield, protein content, and protein yield as well as the dough rheological traits when using genomic estimated breeding values of the traits per se, protein content or the gluten strength and viscosity index for selection.

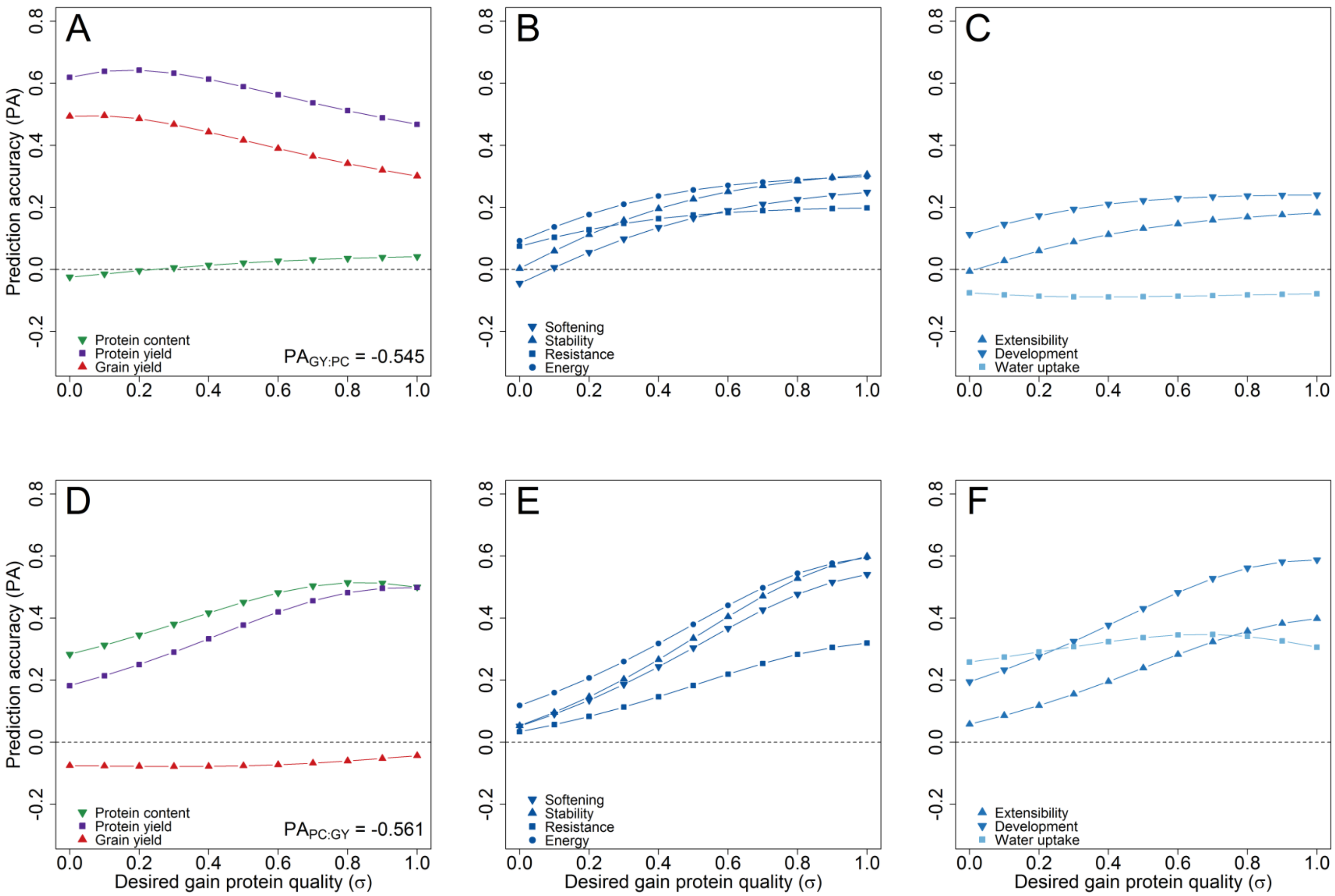

Fig. S5 Prediction accuracy for major agronomic traits, gluten strength and gluten viscosity related dough rheological traits as well as the water uptake with varying desired gains for the protein quality in the grain yield deviation (top row) and grain protein deviation indices (bottom row).

Table S1 Number of pre-selected trials, lines, variation, and entry-mean heritability for grain yield, protein content, protein yield and the assessed dough rheological traits for the phenotypic data from which the 480 lines in the study were extracted

Trait	Trials	Lines	σ_G^2	σ_e^2	h^2	Min	Mean	Max
Grain yield (dt ha^{-1})	136	1838	11.97	34.45	0.61	46.4	66.2	88.8
Protein content (%)	86	1814	0.38	0.40	0.78	10.8	13.7	15.9
Protein yield (dt ha^{-1})	66	1795	0.18	0.73	0.47	6.4	9.3	12.4
Water uptake (%)	28	753	2.76	2.27	0.61	51.7	59.1	64.7
Development (min)	23	603	1.24	2.35	0.41	0.4	4.0	11.1
Stability (min)	23	689	26.15	38.35	0.47	0.2	14.0	31.6
Softening (FU)	25	733	262.58	368.42	0.49	0.8	51.3	175.5
Resistance (EU)	25	747	2966.60	5623.41	0.39	63.2	389.4	715.1
Extensibility (mm)	20	636	130.92	173.69	0.49	120.9	171.1	226.2
Energy (cm^2)	23	716	519.31	341.71	0.66	24.6	114.3	192.3

Genotypic variance (σ_G^2), residual variance (σ_e^2), and heritability (h^2).

Table S2 Variation, genomic heritability, prediction ability and prediction accuracy for grain yield, protein content, protein yield and the assessed dough rheological traits in the investigated population of 480 lines.

					Prediction ability					Prediction accuracy				
Trait	Min	Mean	Max	h^2	MAS (fix)[†]	MAS (random)[‡]	GBLUP[§]	WBLUP (fix)[¶]	WBLUP (random)[#]	MAS (fix)[†]	MAS (random)[‡]	GBLUP[§]	WBLUP (fix)[¶]	WBLUP (random)[#]
Grain yield (dt ha^{-1})	48.2	68.3	83.6	0.60	0.652	0.221	0.223	0.646	0.648	0.259	0.257	0.755	0.748	0.751
Protein content (%)	11.6	13.5	15.9	0.80	0.342	0.336	0.725	0.723	0.724	0.363	0.357	0.769	0.766	0.767
Protein yield (dt ha^{-1})	7.3	9.4	11.4	0.42	0.456	-0.014	-0.005	0.444	0.453	-0.021	-0.008	0.671	0.654	0.667
Water uptake (%)	51.7	58.9	64.7	0.68	0.225	0.219	0.588	0.583	0.587	0.274	0.267	0.728	0.722	0.727
Development (min)	0.6	4.0	11.1	0.49	0.324	0.319	0.565	0.562	0.564	0.418	0.411	0.727	0.723	0.726
Stability (min)	0.3	13.1	31.6	0.44	0.410	0.410	0.506	0.515	0.518	0.589	0.588	0.725	0.723	0.726
Softening (FU)	0.8	52.9	175.5	0.61	0.454	0.455	0.609	0.611	0.613	0.534	0.535	0.717	0.720	0.722
Resistance (EU)	135.5	394.5	715.1	0.53	0.309	0.305	0.437	0.477	0.478	0.454	0.449	0.646	0.704	0.707
Extensibility (mm)	120.9	170.5	224.1	0.53	0.239	0.238	0.544	0.545	0.545	0.309	0.307	0.702	0.703	0.703
Energy (cm^2)	24.6	115.1	192.3	0.65	0.471	0.469	0.583	0.611	0.611	0.588	0.585	0.727	0.763	0.762

[†] Linear model for marker-assisted selection with fixed *Glu-1* marker effects
[‡] Linear model for marker-assisted selection with random *Glu-1* marker effects
[§] Genomic best linear unbiased prediction model
[¶] Weighted genomic best linear unbiased prediction model with fixed *Glu-1* marker effects
[#] Weighted genomic best linear unbiased prediction model with random *Glu-1* marker effects
